# Effect of *Fusarium proliferatum* infection on physiological, phytochemical, and nutrient responses in garlic

**DOI:** 10.7717/peerj.19601

**Published:** 2025-06-20

**Authors:** Kamil Sarpkaya

**Affiliations:** Engineering of Forestry/Faculty of Forestry, University of Karabuk, Karabuk, Turkey

**Keywords:** *Fusarium proliferatum*, Garlic (*Allium sativum*), Physiologic responses, Phenolic compounds, Antioxidant activity

## Abstract

*Fusarium* species are significant pathogens in many crops, including garlic (*Allium sativum*), threatening yield and food safety through mycotoxin production. This study investigates the physiological, phytochemical, and nutrient responses of garlic genotypes (Local-Konya, Babaeski-Kırklareli, and Iranian-Balıkesir) to *Fusarium proliferatum* infection. Phenolic compounds, antioxidant activity, protein content, and macro- and microelement levels were assessed in healthy and infected garlic genotypes. Infection by *F. proliferatum* led to a significant increase in phenolic compounds, especially resveratrol and catechin. The Iranian-Balıkesir genotype exhibited the highest response, showing a 110.9% rise in total phenolic content. Regarding antioxidant activity, 2,2-Diphenyl-1-picrylhydrazy (DPPH) inhibition also rose in all genotypes with the rate of 41.57–55.5% in diseased groups in comparison with healthy groups. However, the protein content of garlic was declined by infection of *F. proliferatum* in all genotypes. Elemental analysis revealed that there were notable drops in potassium and calcium levels, particularly in Local-Konya genotype, but the other elements in plants either increased or decreased accordingly. It was observed that garlic genotypes responded differently to *F. proliferatum* infection in organic acid components. These findings highlighted that *F. proliferatum* infestation in garlic enhanced phenolic production and antioxidant activities as a defense mechanism, but the amount of nutrient content of plants according to fertilization will also affect developing resistance to disease physiologically.

## Introduction

*Fusarium* represents one of the fungi with hosts in the widest range, causing diseases in plants, humans, and animals. Besides, it also produces mycotoxins in its hosts, which bring huge threats to food safety. Recently, it has been noticed that the species of *Fusarium* cause serious problems even in garlic (Dean et al., 2012; Summerell, 2019). *Fusarium proliferatum* has been described as the most common species causing *Fusarium* basal root rot among *Allium* species and is generally associated with *F. oxysporum, F. solani, F. acuminatum, F. redolens, F. verticillioides, F. equiseti, F. culmorum, F. falciforme,* and other species of *Fusarium* (Le, Audenaert & Haesaert, 2021). Liamas et al. (2013) presented *F. proliferatum* as the etiological agent of the postharvest disease and dry rot of garlic during long-term storage. In fact, *F. proliferatum* infection was found to accompany *F. oxysporum* and jointly cause basal rot of the garlic bulbs. More recently, this organism has been reported to cause postharvest dry rot, resulting in losses as high as 30% in yields throughout the world. The rotten condition that results in the principal causes of loss in quality of garlic bulbs during storage is related to practices in postharvest handling such as drying, storage, transportation, and marketing (Mondani et al., 2021).

Various phytohormones, phytochemicals, and peptide hormones participate in plant defense through alterations in their respective expression levels to impede pathogen colonization during infection (Adie et al., 2007; Bari & Jones, 2009). Salicylic acid (SA), ethylene (ET), and jasmonic acid (JA) are the major important plant defense-controlling hormones against *F. oxysporum* disease. The interactions between *Arabidopsis*—*F. oxysporum* f.sp. *conglutinans* and *F. oxysporum* f.sp. *lycopersici* were estimated by Berrocal-Lobo & Molina (2004). By using several mutants impaired in the ET, JA and SA signaling they noticed that a positive cooperation among SA, JA, and ET is needed to assure an effective plant resistance against the evaluated pathogen. SA content in tobacco plants is increased in post infection of *F. solanii* (Luu et al., 2015). Similarly, representatives of the genus *Fusarium* can promote the biosynthesis of phenolic compounds such as chlorogenic acid, caffeic acid, and resveratrol, well known for their abilities to strengthen cell walls and reduce oxidative stress in plants. These phytochemicals have a very vital role in limiting the damage caused by *Fusarium* and further prevent the spread of the pathogen.

In addition, significant responses of plant nutrient content to *Fusarium* infection have been reported. Lobna et al. (2017) observed a significant decrease in the contents of Cu, Zn, Fe, Mn, Mg, and K in the plants of tomato due to co-inoculation with *F. oxysporum* f. sp. *radicis lycopersici* and *Meloidogyne javanica*. On the other hand, Ca content in tomato root significantly increased. Resistance of plants to pathological organisms can be partial or systemic; it may provide fast and gradual physiological changes in plants and nutrient metabolism, for example, changes in root nutrient uptake and photosynthesis in leaf after infection. Nutrient redistribution is quite common for infected plants, which often redirect nutrients for defense at the expense of growth, thus causing nutrient imbalances of critical macro and microelements such as nitrogen, phosphorus, potassium, and calcium (Nadeem et al., 2018).

Moreover, some common physiological symptoms related to *Fusarium* infection include physiological changes such as protein degradation and increased markers of oxidative stress. Among the major physiological markers of resource reallocation by the plant toward defense are reduced protein synthesis and increases in antioxidant activities and scavenging of reactive oxygen species (ROS). These physiological and biochemical changes therefore reflect a complex interrelationship among *Fusarium* infection, phytochemicals production, and nutrient content change. Despite considerable studies on *Fusarium*, very little information is available on the physiological, phytochemical, and nutrient-based responses of garlic after *Fusarium* infection. This study deals with the investigation of *F. proliferatum* infection effects on the production of phenolic compounds, antioxidant activity, protein content, macro-, and microelements of three different garlic genotypes. This work aims to explain the changes in these parameters due to *F. proliferatum* infection and outlines the defensive mechanisms that are switched on within garlic plants.

## Materials & Methods

### Plant and fungal materials

Three garlic genotypes from different regions of Türkiye, where garlic has been grown for a long time, were studied. Plant samples were collected from garlic common storages, which were owned by garlic growers, in August–September 2022. Sampling method was done according to Mondani et al. (2021). Garlic genotypes were harvested at the end of July 2022, when plants reached their physiological maturity. After harvest, garlic were sundried on field ground for two weeks, then garlic bulb samples representing three different genotypes were collected from a total of 13 storage facilities across three provinces in Türkiye. Specifically, 64 bulbs of the Local-Konya genotype were obtained from five storage houses located in Konya, 46 bulbs of the Babaeski-Kırklareli genotype were collected from six storage houses in Kırklareli, and 49 bulbs of the Iranian-Balıkesir genotype were sourced from two storage houses in Balıkesir. Details about location, genotype characteristics and number of surveyed storages and samples were given in Table 1.

**Table 1:** Location, GPS (global positioning system) data and genotypes characteristics of plant and fungal materials.

**Provinces**	**Altitude (m)**	**Genotype name**	**Genotype characteristics**	***Fusarium proliferatum*** **NCBI Numbers**	**Number of surveyed garlic storages**	**Number of samples for pathogen isolations**	**Number of samples infected with** ***F. proliferatum***	**Disease** **ratio (%)**
Balıkesir	231	Iranian-Balıkesir	Hardneck, White colored, Larger bulbs	JQ762611.1	2	49	1	2,04
Konya	1047	Local-Konya	Hardneck, White colored, Medium sized bulbs, Long-term storage	MT095058.1	5	64	2	3,13
Kırklareli	314	Babaeski-Kırklareli	Hardneck, White colored, Smaller bulbs	MH383509.1	6	46	1	2,17
	TOTAL				13	159	4	2,52

The disease ratio (%) was calculated by dividing the number of garlic bulbs identified as infected with *F. proliferatum* by the total number of bulbs examined for each genotype and then multiplying the result by 100. This value reflects the proportion of visibly or molecularly confirmed infected samples within each genotype group.

The basal part of garlic bulbs was used for the isolation of *F. proliferatum*. The disease exhibiting garlic cloves were first washed under tap water and the small explants containing symptomatic tissue were cut into 0.5–1 cm pieces with the help of sterile scalpel. Explants were soaked in 1.5% NaOCl solution (0.5 mL L^−1^, Tween 20 added) for 5 min in a laminarflow cabinet in order to surface sterilization. For removing sterilant, they were rinsed with sterile distilled water 3 times. Then, they were put in sterile filter papers for 4–5 h to dry (Altınok & Can, 2010). Explants were cultured to Potato Dextrose Agar (PDA) and *Fusarium* Minimal Media (FMM) containing antibiotics (Stretomycin Sulphate, 100 µg mL L^−1^) and incubated 24 ± 2 °C for 5–7 days. At the end of incubation, the fungal colonies developing around the plant tissues were purified and single spore isolation was done.

For the purpose of *F. proliferatum* characterization, single spored isolates were morphologically investigated according to the species identification key of Leslie & Summerell (2006). The characteristics of the colonies were analyzed after the isolates were sub-cultured onto Potato Sucrose Agar (PSA) to determine colony morphology and onto Synthetic Nutrient Agar (SNA) to investigate conidial structures according to Altınok & Can (2010).

In the molecular identification of the fungal isolates, translation elongation factor 1-α (Tef-1α), and RNA polymerase second large subunit (RPB2) regions were used (Dedecan et al., 2022).

After isolations and molecular diagnosis, garlic bulbs without any fungal development were considered as healthy and constituted the healthy garlic group in subsequent biochemical analyses, while the cloves from which *F. proliferatum* isolates were obtained formed the diseased group.

### Total phenolic content extraction and measurement

The extraction of the phenolic compounds was done by homogenizing 10 g garlic cloves in 80 mL of 80% methanol solution using a high-speed homogenizator (IKA™, T25).The homogenate was stored at room temperature in the dark for 24 h and later centrifuged for 10 min at 4,000 g. The resulting supernatant was filtered to discard impurities, and total phenolic content was quantified using the Folin-Ciocalteu method. Absorbance of the resulting solution was measured at 765 nm. The total phenolic content was expressed as mg GAE g^−1^ fresh weight, using a calibration curve prepared with standard gallic acid solutions (Erol, 2024a).

### Extraction and 2,2-Diphenyl-1-picrylhydrazyl (DPPH) free radical scavenging method

For antioxidant activity, garlic cloves were extracted with 85% ethanol in an amount equal to two g. Homogenization was done for samples collected and kept away from light at room temperature for a period of 24 h. Then, centrifugation was done for 10 min with 5,000 g, after which solution was obtained for extraction. The 2,2-Diphenyl-1-picrylhydrazy (DPPH) radical scavenging activity was carried out using the procedure previously described with some modifications. To this end, 0.1 mM DPPH radical was reconstituted in methanol and 100 µL of the extract from each sample was added to three mL of the DPPH solution. The resulting mixture was thereafter kept in the dark for 30 min. The absorbance was consequently measured at an absorbance wavelength of 517 nm using a spectrophotometer while ascorbic acid was used as the positive control. The DPPH radical scavenging capacity was calculated using % inhibition formula according to Ozdenefe et al. (2024).

(1) \begin{eqnarray*}\%\text{Inhibition}=(\text{Absorbance of control}-\text{Absorbance of sample})/(\text{Absorbance of control})\nonumber\\\displaystyle \times 100.\end{eqnarray*}

Assays were done in triplicate, and data are expressed as mean ± standard deviation (SD). Data were analyzed by analysis of variance (ANOVA), and a probability of less than 0.05 was considered significant.

### Protein analysis

The protein content in garlic cloves was determined by the Kjeldahl method. A 0.25 g sample was digested with sulfuric acid (H_2_SO_4_) and a catalyst, with combustion for 3.5 h. The digested solution was then distilled in an alkaline environment created by 40% sodium hydroxide, NaOH, and the ammonia collected with 4% boric acid. The distillate obtained was then titrated against 0.1 N hydrochloric acid (HCl) for protein content. The calculations were performed according to the guidelines laid out by Casado et al. (2004).

### Mineral content analysis

The samples of garlic cloves were dried at 72 °C and ground into powder form for the analysis of their mineral content. A 0.25 g portion of each sample was then subjected to digestion using a mixture of nine mL nitric acid (HNO_3_) and three mL hydrogen peroxide (H_2_O_2_) in a microwave digestion system operating with 200 W for 30 min. The digested samples were filtered and diluted to a final volume of 25 mL. The concentration of Ca, Mg, Fe, Mn, Zn, and Cu was done by atomic absorption spectroscopy, while for Na and K, flame photometry was done (Besirli et al., 2022). The level of N and P was determined in a UV spectrophotometer. All the measurements were carried out in three times for reliability as technical replicates (triplicates).

### Extraction method and high-performance liquid chromatography (HPLC) analysis conditions of phenolic compounds

The phenolic compounds were extracted from garlic cloves using a method optimized for maximum recovery. About 10 g of each garlic sample was homogenized in 80 mL of 80% methanol and incubated at room temperature in the dark for 24 h. On incubation, the mixture was centrifuged at 4,000 g for 10 min to separate the supernatant. Filtration was subsequently carried out on the supernatant to remove impurities and improve the solubility of the phenolic compounds (Erol, 2024b).

The separation and quantification of phenolic compounds were performed on a reverse phase high-performance liquid chromatography (RP-HPLC) system fitted with a C18 column (4.6 × 250 mm, five µm particle size). The column temperature was kept at 30 °C, and a diode array detector (DAD) was set to monitor the phenolic peaks. A gradient elution system was performed when using two mobile phases: A, 0.1% phosphoric acid in water, and B, 100% acetonitrile. The volume of injection was 10 µL, and the flow rate was one mL/min. Identification and quantification of phenolic compounds were performed in accordance with comparison of retention times with those of external standards. Firstly, standard solutions with known concentrations of phenolic compounds were plotted in calibration curves. Results were given as milligrams of phenolic content per kilogram of garlic weight. All analyses were run in triplicate in order to assure precision and repeatability (Erol, 2024b).

### Extraction method and HPLC analysis conditions of organic acids and sugars

Organic acids and sugars were extracted from garlic cloves. Approximately 2.5 g of finely crushed garlic samples were placed into 50 mL Falcon tubes, to which 25 mL of a deionized water/methanol mixture (7:3, v/v) was added. The mixture was thoroughly homogenized using a high-speed homogenizator (IKA™,T25). The homogenate was then incubated in a water bath at 40 °C for 30 min to aid extraction. The solution was then centrifuged for 10 min at a speed of 10,000 g at 4 °C and the supernatant filtered *via* a 0.45-micron syringe filter. The filtered extracts were kept under −20 °C until further analysis (Erol, 2024b).

Organic acids and sugars in the garlic extracts were determined by a high-performance liquid chromatography system. Separation of sugars was done on a Rezex RCM-Monosaccharide Ca^2^^+^ (8%) LC Column (300 × 7.8 mm). Column temperature was maintained at 80 °C, with analysis done in an isocratic mode by using ultrapure water as a mobile phase at a flow rate of 0.5 mL/min. The analysis was completed within 15 min, and the glucose, fructose, and sucrose concentrations were calculated from calibration curves prepared using standard sugar solutions. Results were presented as milligrams of sugar per gram of fresh weight, and three replicates of each sample were analyzed (Erol, 2024b).

Organically bound acid was determined by the same high-performance liquid chromatography (HPLC) system, only with a different column: Rezex ROA-Organic Acid H^+^ (8%) LC Column (300 × 7.8 mm). The column temperature was 50 °C, and the UV detector was set at 210 nm. A gradient elution system was used for separating a number of organic acids. The analytical results for organic acids were determined using calibration curves built from standard solutions of the target organic acids. Milligrams of organic acid were expressed per gram weight (Erol, 2024b).

### Statistical analysis

All biochemical, elemental, and HPLC analyses were conducted using three biological replicates for each genotypes (for both healthy and diseased samples). Each replicate was prepared by pooling 20 cloves, ensuring representative sampling within the group. The variables that were considered in this correlation analysis including phenolic compounds, organic acids, sugars, antioxidant activity, elements, total phenolic content, and protein levels were calculated using a Pearson’s correlation coefficient. The data set on the analysis of elements was used to construct a correlation matrix by summarizing and visualizing it through a heatmap showcasing relationships as positive or negative. Then, principal component analysis (PCA) was applied to gauge the variation among phenolic compounds, reducing data dimensions into two main components. The distribution of genotypes and their effect regarding *F. proliferatum* infection on the selected phenolic compounds were shown in biplot chart. Correspondingly, changes in the organic acid concentration within different genotypes were compared by applying line charts for healthy and diseased genotypes. Mean comparisons were done by analysis of variance (ANOVA), followed by Tukey’s honestly significant difference (HSD) test to establish the significant difference among the samples. Any results with a *p* ≤ 0.05 were considered significant. Furthermore, PCA and other data visualization techniques were carried out using JMP 14 and OriginPro.

## Results

### Isolation of Fusarium proliferatum from garlic bulbs

**Figure 1:**
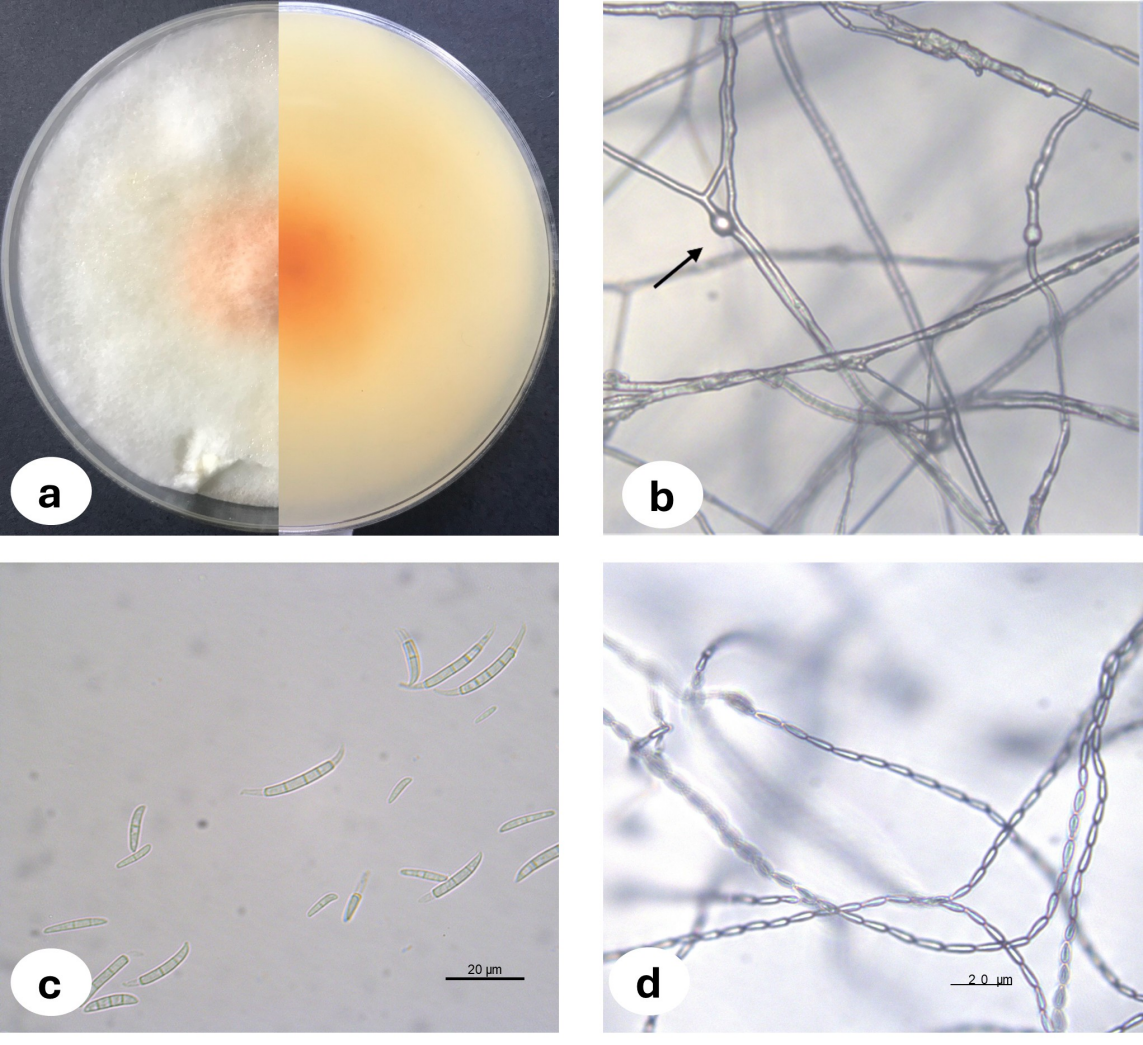
*Fusarium proliferatum* colony morphology on PSA medium (A) chlamidospore (B) macroconidia (C) microconidia in chain (D).

Upon examination of the garlic bulbs collected from storage houses, browning was observed at the basal parts of the cloves, along with rot progressing from the basal area towards the tip. A total of 159 garlic bulbs were studied and 2.52% of the garlic bulbs were classified as infected with *F. proliferatum* where three of the fungal isolates were deposited in the National Center for Biotechnology Information (NCBI) and accession numbers were assigned (Table 1). The morphological characteristics of *Fusarium proliferatum* isolates grown on PSA and SNA media were represented in Fig. 1. For the biochemical analysis, healthy and *F. proliferatum* infected garlic cloves were used.

**Figure 2:**
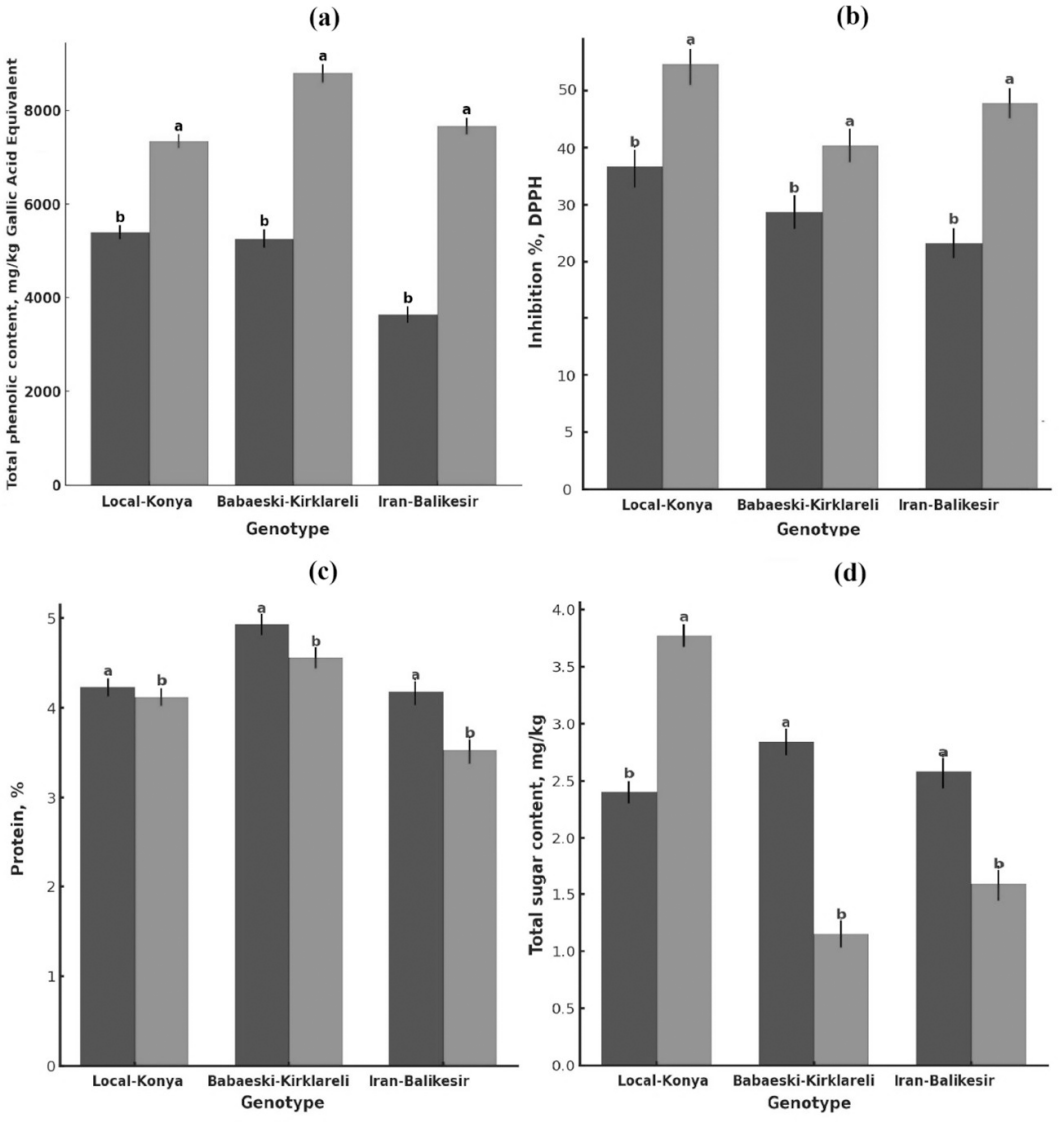
Changes in (A) total phenolic content, (B) antioxidant activity, (C) protein content (mg kg^−1^) and (D) total sugar content of garlic genotypes. Values are mean ± standard deviation. Different letters indicate statistically significant differences (*p* ≤ 0.05). Black, heallthy; grey, diseased.

### Total phenolic contents

Total phenolic content was significantly higher in the infected than in the healthy plants (Fig. 2A, Table 2). Total phenolic content in the Local-Konya genotype of healthy plants was 5,400.96 mg kg^−1^ and increased by 36.0% to 7,347.44 mg kg^−1^ in infected plants (*p* ≤ 0.01). While the phenolic content was 5,264.72 mg kg^−1^ in healthy plants for the Babaeski-Kırklareli genotype, the infection increased it by 66.9% and reached the value 8,790.07 mg kg^−1^ (*p* ≤ 0.001). For the genotype Iranian-Balıkesir, the content of total phenolic was found to be 3,634.33 mg kg^−1^ in healthy plants and increased by 110.9% to 7,664.61 mg kg^−1^ in infected plants (*p* ≤ 0.001). According to the statistical analysis, it was found that the total phenolic compounds increased significantly in all the studied genotypes after *F. proliferatum* infection. It is obvious that the 110.9% increase in the Iranian-Balıkesir genotype might show a meaningful increase in phenolic compound production due to resistance against this disease. Overall, an increase in the total phenolic content was statistically significant (at least at *p* ≤ 0.05) in all studied genotypes.

**Table 2:** Comparison of total phenolic, antioxidant activity, total protein and total sugar content of diseased/healthy garlic genotypes.

	**Total phenolics** **(mg/kg GAE)**	**Antioxidant activity** **(Inhibition, DPPH %)**	**Total protein** **(%)**	**Total sugar (Sucrose, mg kg** ^−1^ **)**
**Disease status (DS)**	^***^	^***^	^**^	^*^
Diseased	7,934.04 A	46.46 A	4.45 A	2.61 A
Healthy	4,766.67 B	34.21 B	4.07 B	2.17 B
**Genotypes (G)**	^**^	^*^	^*^	^*^
Local-Konya	6,374.20 b	45.16 a	4.17 a	3.09 a
Babaeski-Kirklareli	7,027.39 a	37.60 b	4.74 a	1.99 c
Iranian-Balikesir	5,649.47 c	38.25 b	3.85 b	2.08 b
**Mean**	6,350.35	40.34	4.26	2.39
**Standart deviation**	±689.27	±4.19	±0.45	±0.61
***DS x G***	^**^	^*^	^*^	^*^

**Notes.**

Letters show the mean values of different groups in each column.

* *p* ≤ 0.5, ** *p* ≤ 0.01, ***p ≤ 0.001, as indicated by the Tukey’s HSD test (*n* = 3).

### Antioxidant activity

*F. proliferatum* infection enhanced the antioxidant activity of all genotypes (Fig. 2B, Table 2). While the DPPH radical inhibition was 39.07% in healthy plants of the Local-Konya genotype, it increased to 51.25% in the infected plants, reflecting a 31.1% increase (*p* ≤ 0.05). In the genotype Babaeski-Kırklareli, antioxidant activity increased from 33.63% in healthy plants to 41.57% after the infection, marking a 23.6% rise (*p* ≤ 0.05). In the Iranian-Balıkesir genotype, antioxidant activity increased from 29.94% in healthy plants to 46.57% in infected plants, which is a 55.5% increase (*p* ≤ 0.01). Statistical analysis carried out with respect to *F. proliferatum* infection showed that antioxidant activities increased significantly. It can be said that with the increase of 55.5% observed in the Iranian-Balıkesir genotype, this genotype developed a good antioxidant defense against oxidative stress caused by *F. proliferatum* infection. Increases in antioxidant activities in all genotypes were significant statistically at least at *p* ≤ 0.05.

### Total protein content

*F. proliferatum* infection effects on the protein content of garlic plants showed lower protein contents of all genotypes in comparison to that of the healthy ones (Fig. 2C, Table 2). The protein content of healthy plants was 4.23% for the Local-Konya genotype and declined by 2.6% to 4.12% in the infected ones, significantly lower at *p* ≤ 0.05. In the healthy plants of the Babaeski-Kırklareli genotype, protein contents were determined as 4.93%, but it was observed to decrease by 7.5% to 4.56% due to *F. proliferatum* infection (*p* < 0.01). In the Iranian-Balıkesir genotype, it was 4.18% in the healthy plants, while it also decreased by 15.5% in the infected ones as in the first group to 3.53% (*p* < 0.001).

Whereas the reduction in protein content was observed for all genotypes, the Iranian-Balıkesir genotype showed a greater reduction. One-way ANOVA showed that these reductions were at a statistically significant level in all genotypes (*p* ≤ 0.05). Among the genotypes, the highest reduction was obtained in the Iranian-Balıkesir genotype. This result definitely shows that *F. proliferatum* infection may interfere with the synthesis and metabolism of proteins by the plants, hence causing such a sharp loss of the protein content.

### Element analysis

*F. proliferatum* infection caused significant variations in both macro- and microelement levels among all the garlic genotypes tested (Table 3). The nitrogen (N) level was stable for both the healthy and infected Local-Konya plants at 0.81% (*p* > 0.05); regarding phosphorus, it increased by 43.6% to 430.62 mg/100 g (*p* ≤ 0.01). K decreased slightly by 1.4% for 5,607.23 mg kg^−1^, whereas for Ca, there was a 13.3% decrease, which was 1,374.31 mg kg^−1^ (*p* ≤ 0.05). In the Babaeski-Kırklareli genotype, N decreased from 0.96% to 0.91% (*p* ≤ 0.05), while P increased by 10.4% to 477.32 mg/100 g (*p* ≤ 0.01). K dropped by 7.5% to 6,472.62 mg kg^−1^ (*p* ≤ 0.05), and Ca decreased by 16.4% to 1,712.5 mg kg^−1^ (*p* ≤ 0.01). In the Iranian-Balıkesir genotype, N dropped to 0.67% (*p* ≤ 0.05), and K levels fell by 13.9% to 4,798.5 mg kg^−1^ (*p* ≤ 0.01). P levels showed a slight increase to 498.35 mg/100 g (*p* > 0.05), while Ca levels decreased by 24.3% to 1,337.88 mg kg^−1^ (*p* ≤ 0.01). These results highlight that *F. proliferatum* infection caused significant differences in macro element levels between genotypes, particularly in potassium and calcium levels, which showed notable reductions.

**Table 3:** Comparison of macro- and microelements contents of diseased/healthy garlic genotypes.

	**N (%)**	**P (mg 100g** ^−1^ **)**	**K (mg kg** ^−1^ **)**	**Ca (mg kg** ^−1^ **)**	**Cu (mg kg** ^−1^ **)**	**Mn (mg kg** ^−1^ **)**	**Fe (mg kg** ^−1^ **)**	**Zn (mg kg** ^−1^ **)**	**Mg (mg kg** ^−1^ **)**	**Na (mg kg** ^−1^ **)**	**Ni (mg kg** ^−1^ **)**
**Disease status (DS)**	^*^	^*^	^**^	^**^	^**^	^**^	^**^	***ns***	^**^	^**^	^**^
Diseased	0.80 B	468.76 A	5,626.11 B	1,474.90 B	2.29 A	6.33 B	20.06 B	17.62 A	235.44 B	133.37 B	0.012 B
Healthy	0.86 A	408.13 B	6,087.02 A	1,800.10 A	2.18 B	9.28 A	25.01 A	17.91 A	314.58 A	160.89 A	0.080 A
**Genotypes (G)**	^**^	^**^	^**^	^**^	^**^	^**^	^**^	^**^	^**^	^**^	^*^
Local-Konya	0.81 b	365.22 c	5,646.88 b	1,479.89 c	3.09 a	6.15 b	16.29 c	16.75 b	232.27 b	123.46 c	0.045 b
Babaeski-Kirklareli	0.93 a	454.83 b	6,734.55 a	1,880.52 a	2.39 b	11.45 a	30.52 a	21.22 a	352.55 a	175.58 a	0.055 a
Iranian-Balikesir	0.74 c	495.28 a	5,188.27 c	1,552.08 b	1.22 c	5.80 c	20.78 b	15.31 c	240.20 b	142.33 b	0.040 b
**Mean**	0.83	438.44	5,856.57	1,637.49	2.23	7.80	22.53	17.76	275.01	147.13	0.046
**Standart deviation**	±0.10	±66.56	±794.18	±213.54	±0.94	±3.17	±7.27	±3.08	±67.27	±26.39	±0.01
***DS x G***	^**^	^**^	^**^	^**^	^**^	^**^	^**^	^*^	^**^	^**^	^*^

**Notes.**

Letters show the mean values of different groups in each column.

ns, non-significant.

* *p* ≤ 0.5, ** *p* ≤ 0.01, ***p ≤ 0.001, as indicated by the Tukey’s HSD test (*n* = 3).

Regarding microelements, copper (Cu) levels varied significantly between genotypes (Table 3). In the Local-Konya genotype, Cu levels increased by 145.2% from 1.79 mg kg^−1^ in healthy plants to 4.39 mg kg^−1^ in infected plants (*p* ≤ 0.01). In the Babaeski-Kırklareli genotype, Cu levels showed a slight decrease to 2.28 mg kg^−1^, which was not statistically significant (*p* > 0.05). In the Iranian-Balıkesir genotype, Cu levels dropped sharply from 2.24 mg kg^−1^ to 0.20 mg kg^−1^ (*p* ≤ 0.001). Iron (Fe) levels showed a slight increase to 16.7 mg kg^−1^ in the Local-Konya genotype (*p* > 0.05), while they decreased by 52.3% to 13.41 mg kg^−1^ in the Iranian-Balıkesir genotype (*p* ≤ 0.01). Magnesium (Mg) levels decreased across all genotypes, with the largest reduction observed in the Babaeski-Kırklareli genotype, where Mg levels fell by 34.2% to 279.71 mg kg^−1^ (*p* ≤ 0.01). In the Iranian-Balıkesir genotype, Mg levels decreased by 25.6% to 204.97 mg kg^−1^ (*p* ≤ 0.05). These findings indicate that *F. proliferatum* infection significantly affected microelement levels, particularly in copper and iron, with notable differences observed between genotypes.

### HPLC analysis of phenolic compounds

The HPLC analysis of phenolic compounds revealed significant effects of *F. proliferatum* infection on phenolic content across the Local-Konya, Babaeski-Kırklareli, and the Iranian-Balıkesir genotypes (Table 4). In the Local-Konya genotype, chlorogenic acid levels in healthy plants were 210.99 mg kg^−1^, but after *F. proliferatum* infection, they decreased by 29.0% to 149.78 mg kg^−1^ (*p* ≤ 0.05). In the Babaeski-Kırklareli genotype, chlorogenic acid levels dropped by 65.7% from 220.46 mg kg^−1^ in healthy plants to 75.48 mg kg^−1^ in infected plants (*p* ≤ 0.01). Conversely, in the Iranian -Balıkesir genotype, chlorogenic acid increased by 55.7% from 88.35 mg kg^−1^in healthy plants to 137.60 mg kg^−1^ in infected plants (*p* ≤ 0.05). Catechin, which was undetectable in healthy plants, was found to be 276.43 mg kg^−1^ in infected Babaeski-Kırklareli plants (*p* ≤ 0.001). Similarly, catechin levels rose to 252.55 mg kg^−1^ in infected Iranian-Balıkesir plants, where it was also absent in healthy plants (*p* ≤ 0.001).

**Table 4:** Comparison of phenolic compounds of diseased/healthy garlic genotypes.

	**Diseased genotypes**			**Healthy genotypes**			Mean
**Organic acids**	**Local- Konya**	**Babaeski- Kirklareli**	**Iranian- Balikesir**	**Local- Konya**	**Babaeski- Kirklareli**	**Iranian- Balikesir**	
**Chlorogenic acid**	210.99 a	220.46 a	88.35 b	149.78 a	75.48 c	137.60 b	147.110
**Catechin hydrate**	nd	nd	nd	nd	276.43 a	252.55 b	88.163
**Caffeic acid**	nd	0.91 a	0.36 b	3.01 a	1.34 b	1.32 b	1.157
**4-hydroxy benzoic acid**	nd	nd	nd	nd	nd	0.36 a	0.060
**Vanillin**	nd	nd	nd	0.23 a	nd	nd	0.038
**Routine**	nd	nd	nd	nd	nd	nd	0.000
**Trans-ferulic acid**	nd	nd	nd	nd	nd	nd	0.000
**Hydroxy cinnamic acid**	nd	nd	nd	0.69 b	0.67 b	5.19 a	1.092
**Naringin**	nd	2.04 a	0.00	nd	nd	nd	0.340
**Orto-coumaric acid**	nd	0.13 a	0.01 b	nd	0.10 a	0.03 b	0.045
**Rosmarinic acid**	2.23 a	nd	nd	nd	nd	nd	0.372
**Salicylic acid**	nd	nd	nd	nd	nd	nd	0.000
**Resveratrol**	0.05 a	0.04 a	0.04 a	0.14 b	0.17 b	0.46 a	0.153
**Quercetin**	1.23 a	1.89 a	0.00	nd	1.61 b	2.74 a	1.245
**Trans-cinnamic acid**	nd	nd	nd	nd	nd	3.92	0.654
**Naringenin**	nd	nd	nd	nd	nd	nd	0.000
**Chrysin**	3.62 b	28.07 a	0.00	3.62 a	1.47 b	nd	6.632
**Flavones**	nd	nd	nd	4.04 b	9.76 a	nd	2.300
**Mean**	12.118	14.086	4.931	9.288	20.391	22.454	

**Notes.**

Letters indicate the mean values of different groups in each row. nd, not detected (*n* = 3).

Caffeic acid was undetectable in the healthy Local-Konya plants but measured at 3.01 mg kg^−1^ in infected plants (Table 4). In the Babaeski-Kırklareli genotype, caffeic acid levels increased by 47.3% from 0.91 mg kg^−1^ in healthy plants to 1.34 mg kg^−1^ in infected plants (*p* ≤ 0.05). In the Iran-Balıkesir genotype, caffeic acid rose slightly from 0.36 mg kg^−1^ in healthy plants to 1.32 mg kg^−1^ in infected plants (*p* ≤ 0.05). 4-hydroxybenzoic acid was only detected in infected Iranian-Balıkesir plants at 0.36 mg kg^−1^, while it was absent in the other genotypes. Vanillin levels increased to 0.23 mg kg^−1^ in the infected Local-Konya plants (*p* ≤ 0.05), with no significant difference observed in the other genotypes. Rutin levels remained low in the Iranian -Balıkesir genotype and showed no significant changes after *F. proliferatum* infection. T-ferulic acid, which was undetectable in the healthy Babaeski-Kırklareli plants, increased to 0.67 mg kg^−1^ following infection (*p* ≤ 0.01), while remaining at low levels in the other genotypes. Naringin, found at 2.04 mg kg^−1^ in the healthy Babaeski-Kırklareli plants, disappeared in infected plants, while only low levels were detected in the Iranian-Balıkesir genotype. O-coumaric acid levels in the Babaeski-Kırklareli genotype decreased by 23.1%, from 0.13 mg kg^−1^ in healthy plants to 0.10 mg kg^−1^ in infected plants (*p* ≤ 0.05), while in the Iranian -Balıkesir genotype, this compound showed a slight increase to 0.03 mg kg^−1^ following infection (*p* ≤ 0.05).

Resveratrol levels increased dramatically in the Iranian-Balıkesir genotype, rising by 998.5% from 0.042 mg kg^−1^ in healthy plants to 0.461 mg kg^−1^ in infected plants (Table 4). A significant increase in resveratrol levels was also observed in the Local-Konya genotype, from 0.051 mg kg^−1^in healthy plants to 0.144 mg kg^−1^ in diseased plants, representing a 182.4% increase (*p* ≤ 0.01). Quercetin levels in the Babaeski-Kırklareli genotype decreased by 14.8%, from 1.89 mg kg^−1^in healthy plants to 1.61 mg kg^−1^in infected plants (*p* ≤ 0.05), with no significant changes in quercetin levels observed in the other genotypes. These results indicate differential responses in the biosynthesis of phenolic compounds by garlic plants according to their genotype after *F. proliferatum* infection. While in some phenolic compounds, such as resveratrol and catechin, significant enhancements were observed, in others, such as quercetin and O-coumaric acid, there was a reduction. Most of these changes were statistically significant (*p* ≤ 0.05) by statistical analysis. A strong defense response can be seen in compounds such as resveratrol against *F. proliferatum* infection.

### HPLC analysis of organic acids and sugars

**Figure 3:**
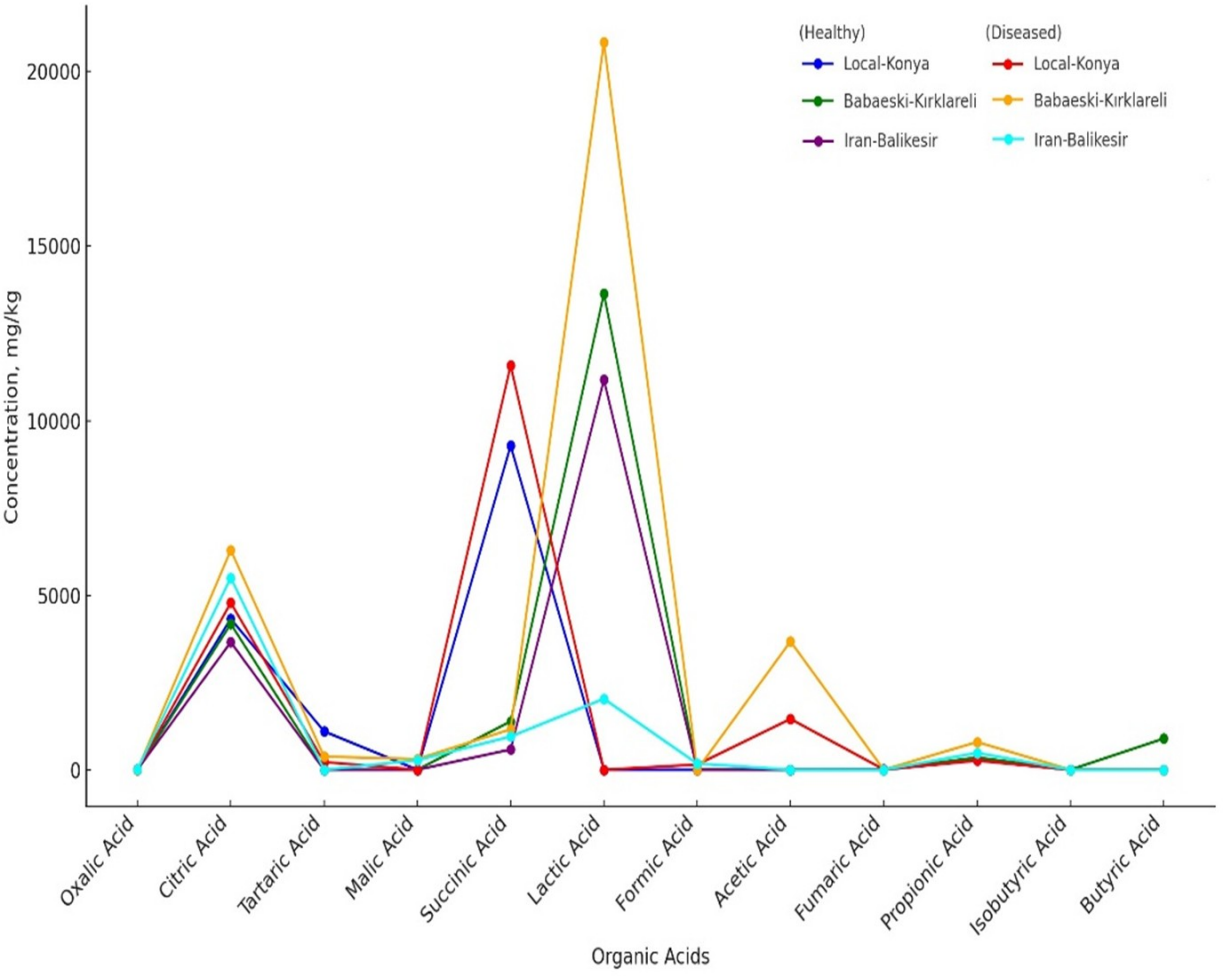
Organic acids of healthy and diseased garlic genotypes. Values are mean ± standard deviation.

Organic acid profiles determined by HPLC analysis showed that *F. proliferatum* infection significantly affects garlic genotypes (Table 5, Fig. 3). The oxalic acid level increased from non-detected in healthy Local-Konya plants to 15.17 mg kg^−1^ in infected plants. For the Iranian-Balıkesir genotype, too, the oxalic acid level increased from 7.01 mg kg^−1^ in healthy plants to 10.56 mg kg^−1^ in infected ones (*p* ≤ 0.05). Citric acid levels in the Babaeski-Kırklareli genotype increased from 4,175.95 mg kg^−1^ in healthy plants to 6,291.02 mg kg^−1^ following infection (*p* ≤ 0.01). In the Local-Konya genotype, citric acid levels rose from 4,322.72 mg kg^−1^ in healthy plants to 4,781.87 mg kg^−1^ in infected plants (*p* ≤ 0.05). Succinic acid showed significant increases in all genotypes, especially in the Local-Konya plants, where it rose from 9,281.79 mg kg^−1^ in healthy plants to 11,579.81 mg kg^−1^ in infected plants (*p* ≤ 0.01).

**Table 5:** Comparison of organic acid compounds of diseased/healthy garlic genotypes.

	**Diseased genotypes**			**Healthy genotypes**			Mean
**Organic acids**	**Local- Konya**	**Babaeski- Kirklareli**	**Iranian- Balikesir**	**Local- Konya**	**Babaeski- Kirklareli**	**Iranian- Balikesir**	
**Oxalic acid**	nd	4.01 b	7.01 a	15.17 a	nd	10.56 b	6.12
**Citric acid**	4,322.72 a	4,175.95 b	3,654.26 c	4,781.87 c	6,291.02 a	5,498.69 b	4,787.41
**Tartaric acid**	1,100.43 a	nd	nd	219.92 b	377.59 a	nd	282.99
**Malic acid**	nd	nd	nd	nd	305.87 a	274.54 b	96.73
**Succinic acid**	9,281.79 b	1,386.63 a	580.82 c	11,579.81 a	1,159.36 b	951.96 c	4,156.73
**Lactic acid**	nd	13,639.16 a	11,168.82 b	0.00	20,840.81 a	2,031.90 b	7,946.78
**Formic acid**	nd	nd	nd	147.37 a	0.00	176.03 a	53.90
**Acetic acid**	nd	nd	nd	1,455.32 b	3,675.93 a	nd	855.21
**Fumaric acid**	5.18 a	nd	nd	8.02	nd	nd	2.20
**Propionic acid**	292.11 b	353.52 a	262.93 c	272.63 c	795.44 a	483.47 b	410.02
**Isobutyric acid**	nd	nd	nd	nd	nd	nd	0.00
**Butiric acid**	nd	892.23 a	nd	nd	nd	nd	148.70
**Mean**	1,250.19	1,704.29	1,306.15	1,540.01	2,787.17	785.60	

**Notes.**

Letters indicate the mean values of different groups in each row. nd, not detected (*n* = 3).

Among other organic acids, malic acid was not detected in healthy Babaeski-Kırklareli plants but measured at 305.87 mg kg^−1^ in infected plants (*p* ≤ 0.05). Lactic acid levels also increased significantly; in the Babaeski-Kırklareli genotype, levels rose from 13,639.16 mg kg^−1^ in healthy plants to 20,840.81 mg kg^−1^ in infected plants (*p* ≤ 0.01), while in the Iranian-Balıkesir genotype, lactic acid increased to 2,031.90 mg kg^−1^ in infected plants.

The sugar analysis revealed that *F. proliferatum* infection had significant effects on sucrose levels (Table 2, Fig. 2D). In the Local-Konya genotype, sucrose levels increased by 57.2% from 2.40 mg kg^−1^ in healthy plants to 3.77 mg kg^−1^ in infected plants (*p* ≤ 0.01). However, in the Babaeski-Kırklareli genotype, sucrose levels dropped by 59.5%, from 2.84 mg kg^−1^ in healthy plants to 1.15 mg kg^−1^ in infected plants (*p* ≤ 0.05). Similarly, in the Iranian-Balıkesir genotype, sucrose levels decreased by 38.5%, from 2.58 mg kg^−1^ in healthy plants to 1.59 mg kg^−1^ in infected plants (*p* ≤ 0.05). These results suggest that *F. proliferatum* infection influences the synthesis of organic acids and sugars differently depending on the genotype. Valuably enough, the increased levels detected at succinic acid, lactic acid, and sucrose upon infection might suggest providing a more direct indication of the metabolic response of the plant. Most of these changes have already been statistically proven (*p* ≤ 0.05) to be significant.

### Multivariate analysis

**Figure 4:**
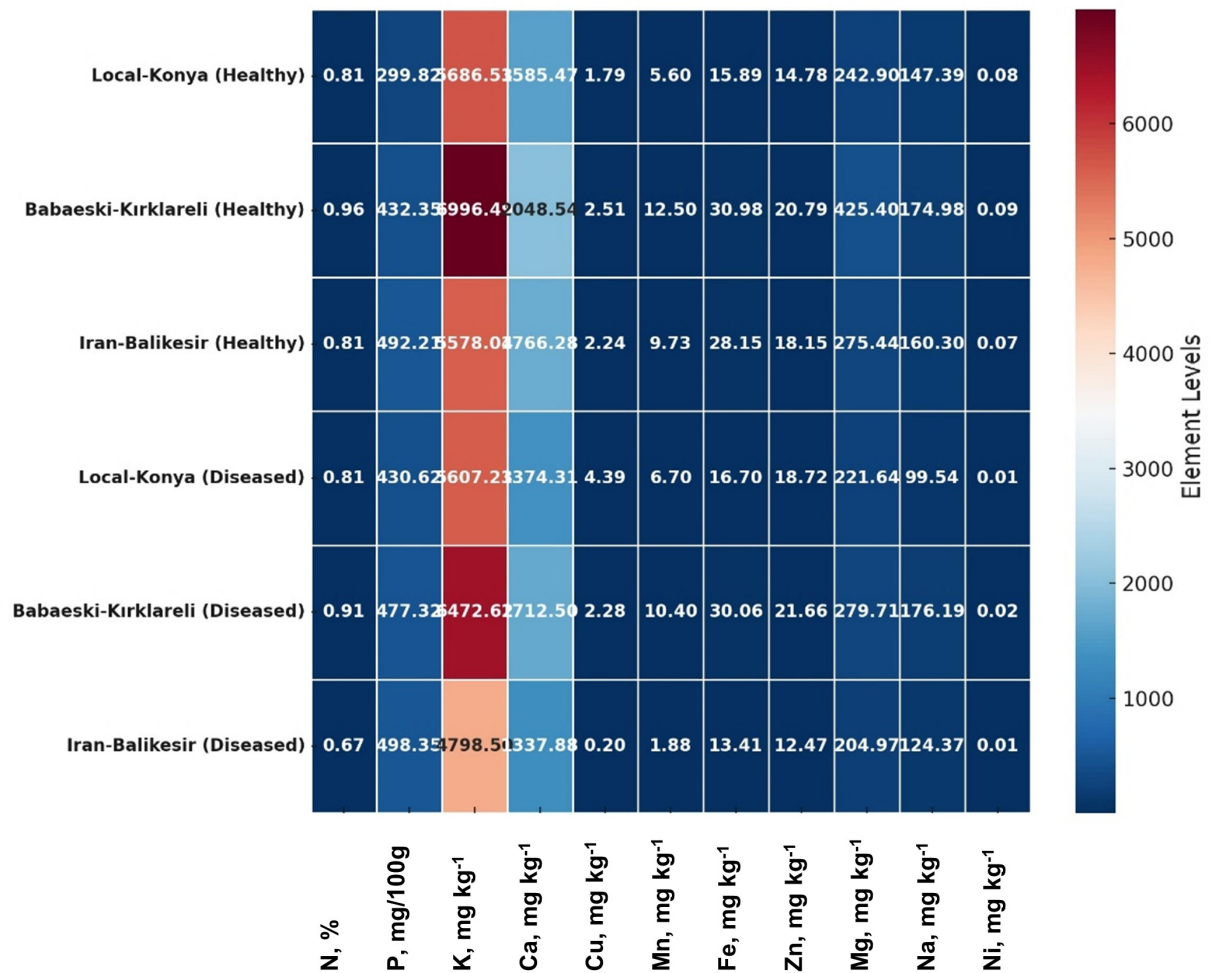
Heatmap plot of macro- and microelements of healthy and diseased garlic genotypes. Values are mean ± standard deviation.

Multivariate analyses were performed in order to obtain an overview of the interactions among the parameters studied, garlic genotypes and the pathogen. The heatmap very well demonstrated great changes between the healthy and diseased plants in a level of both macro- and microelements (Fig. 4), where red color indicates a higher and blue lower level, thus showing great differences among genotypes. In the case of the Local-Konya genotype, a significant decline in Ca and K levels can be observed between healthy and diseased plants. With respect to the Babaeski-Kırklareli genotype, high levels of elements in the healthy plants dropped to low values when under *F. proliferatum* infestation. In diseased plants, a serious decrease of copper, along with other microelements, was observed in the Iranian-Balıkesir genotype. The Ni levels were significantly reduced at *p* ≤ 0.001, hence showing a great deal of difference between healthy and diseased plants. Overall, this heatmap clearly presented the consequence of *F. proliferatum* infection on plant nutrient levels and the importance of such changes.

**Figure 5:**
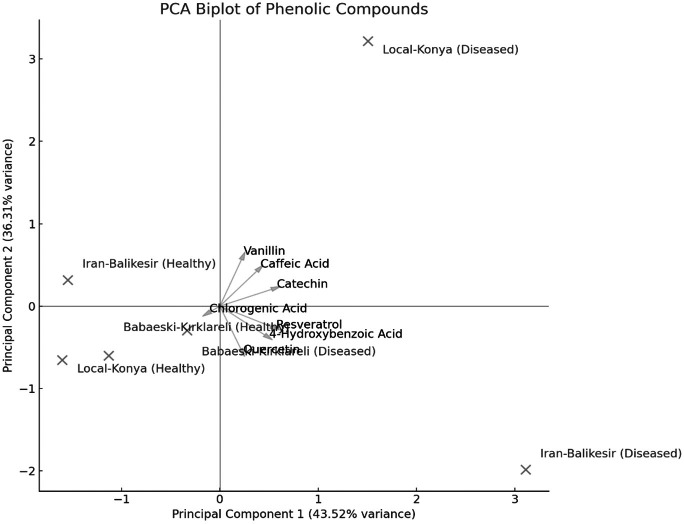
Principal component analysis (PCA) results of the phenolic contents of diseased and healthy genotypes.

The result of PCA biplot analysis (Fig. 5) illustrates differentiation among genotypes because of *F. proliferatum* infection, as explained by two major principal components. Notably, in both healthy and diseased plants, the Local-Konya genotype clustered closely, indicating minimal changes in phenolic compound levels. On the other hand, the Babaeski-Kırklareli genotype showed clear differentiation between healthy and diseased plants. Among them, the variables chlorogenic acid, caffeic acid and resveratrol contributed a lot to genotype separation, whereas catechin and quercetin showed the highest variance especially under disease. The first principal component explained 58.9% of total variance, while the second principal component accounted for 24.5%, which indicates an increased production of these compounds upon *F. proliferatum* infection. Overall, this biplot effectively showed the variation in the effect of *F. proliferatum* infection on phenolic compounds regarding the variability among the genotypes.

**Figure 6:**
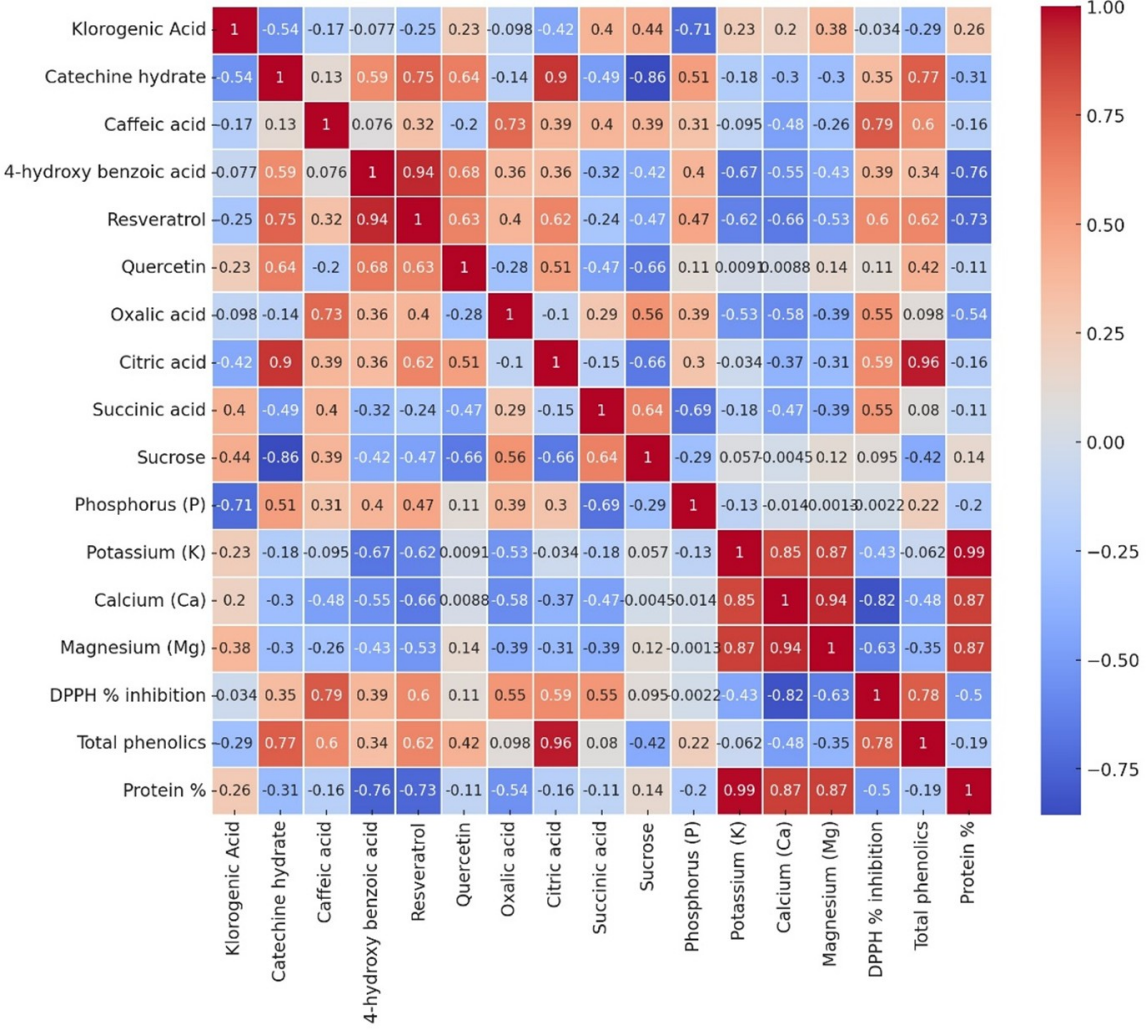
The Pearson correlation analysis results of variables in all garlic genotypes. Each cell depicts the value of the correlation coefficient (r) between two components and is positive or negative. The colors red and the shade of red represent positive correlations with parameters, while blue and the shades of blue show negative ones.

Analysis provides some relationships of all the measured parameters, across three genotypes (Local-Konya, Babaeski-Kırklareli, and Iran-Balıkesir) (Fig. 6). In most cases, it was a positive correlation for the phenolic compounds and antioxidant activity. For instance, strong correlations were observed for compounds such as resveratrol and DPPH % inhibition. A weaker correlation was observed not only between the total phenolic content and protein level, but also within organic acids of the genotypes studied. In terms of elements, potassium (K) had a good positive relationship with some macro elements, especially in magnesium (Mg) and calcium (Ca).

## Discussion

The phenolic compounds are very significant to plants through their intervening roles in the defense mechanisms against the pathogen and stress conditions. In this study, *F. proliferatum* infection was found to increase the total phenolic content in the garlic plants studied. For example, in the Local-Konya genotype (Table 4), the total phenolic contents rose by 36% from 5,400.96 mg kg^−1^ in healthy plants to 7,347.44 mg kg^−1^ in infected plants (*p* ≤ 0.01). Present findings give a signification of the paramount importance of phenolic compounds in plant defense systems against pathogens. The literature shows that phenolic compounds strengthen cell walls and reduce the oxidative stress by scavenging the reactive oxygen species. The role of phenolic compounds in plants for defense against biotic and abiotic stresses was widely reviewed by Tuladhar, Sasidharan & Saudagar (2021). Moreover, according to Miedes et al. (2014), phenolics were involved in lignin synthesis that reinforces cell walls against the penetration of the pathogen. Increased total phenolic content suggests that plants enhance the biosynthesis of secondary metabolites as a result of their reaction against the infectious agent. In connection with this, Tuladhar, Sasidharan & Saudagar (2021) have also found that there has been enhanced biosynthesis of phenolics in plants treated under *Fusarium* pathogens. Some phenolic compounds have a very significant role in plant defense; these prevent pathogen attack through promoting lignin synthesis and cell wall thickening. In this context, Tak & Kumar (2020) reported that biosynthesis of phenolic compounds is induced through pathogen attack and in some cases is effective in counteracting pathogen attack.

Increased antioxidant activity was observed in all genotypes following *F. proliferatum* infection (Table 2). Antioxidants help protect plants from oxidative stress in defense mechanisms. The DPPH radical inhibition in plants of the Iranian-Balıkesir genotype increased by 55.5%, from 29.94% in healthy plants to 46.57% in infected plants; the change was statistically significant (*p* ≤ 0.01). This probably means that under the influence of *F. proliferatum* infection, defense against oxidative stress in plants has increased due to better antioxidant defense. It was further said that oxidative stress causes the initiation of plant defense responses against pathogens and the role in this process is being taken by antioxidant enzymes (Biswas et al., 2020). Suman et al. (2021) elaborated that antioxidants act to protect the plant cell under oxidative stress by neutralizing ROS. The increase in antioxidant activity as found in this study means that the plant produces the antioxidant compound is to neutralize ROS in the defense response of pathogens. Chrpová et al. (2021) therefore said that infection from *Fusarium* species would promote increased ROS generation in the plants, and they in turn activated their antioxidant defense systems.

*F. proliferatum* infection had a negative effect on the protein synthesis of garlic plants, as their protein levels were reduced in all the tested genotypes (Table 2). In the Iranian-Balıkesir genotype, protein content was reduced by 15.5%, from 4.18% in healthy plants to 3.53% in infected plants (*p* ≤ 0.001). This shows that *F. proliferatum* infection suppresses plant metabolism and protein synthesis. Literatures show that pathogens have negative effects on the protein synthesis in plants, which thereby shifts energy and other resources towards defense responses. Ferreira et al. (2007) reported that the pathogen attacks suppress protein synthesis in plants with the activation of a series of defense responses. Similarly, Dutta et al. (2023) found that the soil-borne pathogens, *Fusarium*, negatively affected protein synthesis within the plants by disrupting metabolic processes. This reduction in protein synthesis may indicate that plants utilize their energy resources in growth and development to incorporate defense mechanisms. Matyssek et al. (2012) stated that plants, during pathogen attacks, utilize their energy in defense mechanisms instead of growth-related metabolic activities. Rojas et al. (2014) further explained the relationship between reduced protein synthesis and the plant defense response by indicating energy utilization is altered during pathogen defense.

Infection with *F. proliferatum* significantly altered the contents of both macro- and microelements in garlic plants (Table 3). Considering the macroelements, the most serious changes took place according to the concentration of phosphorus and potassium. In the Local-Konya genotype, P increased by 43.6% from 299.82 mg/100 g in healthy plants to 430.62 mg/100 g in infected plants (*p* ≤ 0.01). That might indicate that infected plants require more phosphorus to increase energy production as a result of pathogen infestation. Ahanger et al. (2016) pointed out that P is one of the main mineral elements used in energy metabolism and for proper plant development. Lower values of potassium, on the other hand, are indicative of an impaired water balance and ion exchange post-infection, since potassium is considered a very important action for the health of the plant. Its reduction during the infection impairs the plant mechanism for the maintenance of water balance. Sardans & Peñuelas (2021) reported that potassium maintains water balance and stomatal action in plant cells. Moreover, potassium deficiency reduces vigor in plants and their resistance to pathogenic attacks. It has been documented that potassium-starved plants are highly susceptible to different pathogenic agents due to disturbances of ion balance and defense mechanisms against *Fusarium* spp (Tripathi et al., 2022).

Regarding microelements (Table 3), significant changes were observed in the levels of copper (Cu) and iron (Fe). Copper levels increased by 145.2% in the infected plants as compared to those in the healthy plants of the Local-Konya genotype, representing 1.79 mg kg^−1^ and 4.39 mg kg^−1^, correspondingly (*p* < 0.01). This is an indication that copper is very vital in the plant’s mechanisms of defense against oxidative stress and pathogens. Copper, as a cofactor of antioxidant enzymes, plays a crucial role in the defense against oxidative stress. According to Liu et al. (2018), copper considerably contributes to a decline in oxidative stress and scavenging of ROS in plants. Further, copper contributes to the synthesis of lignin, which strengthens plant cell walls against the penetration of the pathogen (Liu et al., 2018). Notably, significant changes were also observed in iron (Fe) levels. Within the Iranian-Balıkesir genotype, iron decreased by 52.3%, from 28.15 mg kg^−1^ in healthy plants to 13.41 mg kg^−1^ in infected plants (*p* ≤ 0.01). Iron deficiency adversely affects chlorophyll synthesis and photosynthetic activity, leading to reduced plant growth and development. As stated by Abbas et al. (2021), it plays an important role in the synthesis of chlorophyll, hence in energy production, and its deficiency weakens the plant’s defense capacity. Iron deficiency reduces photosynthetic capacity and thus weakens plant mechanisms set up for defense against *Fusarium*.

The infection of garlic plants with *F. proliferatum* significantly altered the level of phenolic compounds (Table 4). In general, increased levels of phenolic compounds, such as resveratrol and catechin, are indicative of increased synthesis in response to pathogen infection. In the genotype Iranian-Balıkesir (Table 4), the resveratrol increased from 0.042 mg kg^−1^ in healthy plants to 0.461 mg kg^−1^ in infected ones, with an increase rate of 998.5% (*p* ≤ 0.001). Mattio et al. (2020) noted that resveratrol is one of the most important stilbenoids involved in plant defense, while stilbenoids have various other activities against pathogens. The increase in catechin levels is particularly noteworthy. In the Babaeski-Kırklareli genotype, while it was not detected in the healthy plants, the level reached 276.43 mg kg^−1^ in infected plants (*p* ≤ 0.001). Among flavonoids, catechin is one type involved in the plant defense mechanisms against oxidative stress. According to Tuladhar, Sasidharan & Saudagar (2021), catechins enhance the plant defense mechanism against oxidative stress and pathogen attack through their antioxidant properties. In this respect, it has also been observed that during oxidative stress, catechins play a role in neutralizing ROS, thereby enhancing the antioxidant defense systems in plants.

*F. proliferatum* infection significantly affected the organic acids and sugars profile of garlic (Table 5). Some organic acids, like succinic acid and lactic acid, showed great increases with infection. Succinic acid increased from 9281.79 mg kg^−1^ in a healthy plant to 11,579.81 mg kg^−1^ in an infected one with the Local-Konya genotype (*p* ≤ 0.01). Succinic acid is one of the major organic acids participating in plant energy metabolism, thus showing increased energy demand and hence accelerated metabolic processes under infection (Wang et al., 2019). As Müller et al. (2012) pointed out, succinic acid is among those metabolites that afford great importance toward generating energy under stress conditions, considered to have a critical role in mitochondrial energy metabolism. Increased lactic acid points out that metabolic pathways are turned on for enhancing stress tolerance (Moons, 2008). In the case of the Babaeski-Kırklareli genotype, lactic acid values showed an increase from 13,639.16 mg kg^−1^ for healthy plants to 20,840.81 mg kg^−1^ in infected plants (*p* ≤ 0.01). Lactic acid would, therefore, be one of the major intermediates in the process of energy production under the conditions of stress in plants and play a major role in their metabolic response.

The line graph has compared the concentrations of organic acids in both healthy and diseased genotypes (Table 5). A significant difference was observed in the levels of acids such as oxalic acid, citric acid, succinic acid, propionic acid, and lactic acid between healthy and diseased plants. For instance, lactic acid showed its highest concentration in genotype Babaeski-Kırklareli (Diseased), which means a higher response against *F. proliferatum* infection. In the organic acid group, citric acid was highly present in the case of diseased conditions of all genotypes, most especially in the Babaeski-Kırklareli genotype. Some organic acids, such as malic acid and fumaric acid, were detected only in specific genotypes depending on their infection. This finding highlights genetic differences among genotypes and underscores the impact of *F. proliferatum* infection.

Changes in sucrose levels indicate that *F. proliferatum* infection affected carbohydrate metabolism in garlic plants (Table 2, Fig. 2D). In the Local-Konya genotype, sucrose increased by 57.2%, from 2.40 mg kg^−1^ in the healthy plants to 3.77 mg kg^−1^ in the infected plants (*p* ≤ 0.01). This increase might suggest that sucrose is an important energy source for the plant during its strengthened defense mechanisms under these stress conditions. Sucrose is one of the critical carbon sources produced within energy production in plants. As explained by Anisimova et al. (2024), its production increases due to responses of defense. According to Mathan, Singh & Ranjan (2021), sucrose acts as an energy sink during stress, where increased levels are recorded during stress. These results indicated that *F. proliferatum* infection strongly affected the organic acid and sugar metabolisms of the garlic plants, which varied greatly among genotypes. This increase in organic acid levels suggests an accelerated energy production process and a potential defense response in infected plants.

## Conclusions

The present study provides a detailed examination of the biochemical and physiological effects of *F. proliferatum* infection in garlic. The infection resulted in significant changes in total phenolic compound levels, antioxidant activity, protein synthesis, macro- and microelement concentrations, phenolic profiles, organic acid composition, and sugar metabolism. The increase in phenolic compounds and antioxidant activity refers to the enhanced mechanisms of plant defense against pathogens, such as *F. proliferatum*. Moreover, the reduction in protein synthesis and deficiencies in key elements such as potassium and iron indicate that the infection had an adverse impact on the physiological functions of garlic. Furthermore, the profiling of organic acids and sugars demonstrated that *F. proliferatum* infection significantly altered plant energy metabolism in garlic.

Soil-borne diseases, especially *Fusarium* wilt, seriously threaten the yield and quality of economically important crops such as garlic. Knowledge gained from this work is of paramount importance for identifying the genotypes that are resistant to *F. proliferatum* infection, and the mechanisms involved in improving plant defense responses.

The results of this study provide clear evidence that phenolic compounds and antioxidant defense mechanisms play a crucial role in inducing plant resistance against *F. proliferatum*. This highlights the promising potential for biochemical defense strategies to enhance disease management in garlic. Ultimately, the findings of this study serve as a critical framework for promoting sustainable agriculture, fostering the development of crops with improved resistance to pathogens, and refining integrated disease management practices.

## Supplemental Information

10.7717/peerj.19601/supp-1 Supplemental Information 1 Raw dataset of Fusarium

## Additional Information and Declarations

### Data Availability

The following information was supplied regarding data availability: The raw data is available in Table 1.
